# Risk assessment for the transmission of Middle East respiratory syndrome coronavirus (MERS-CoV) on aircraft: a systematic review

**DOI:** 10.1017/S095026882100131X

**Published:** 2021-06-10

**Authors:** T. Berruga-Fernández, E. Robesyn, T. Korhonen, P. Penttinen, J. M. Jansa

**Affiliations:** 1 Department of Medical Biochemistry and Microbiology (IMBIM), Uppsala University, Uppsala, Sweden; 2 Emergency Preparedness and Response Support, European Centre for Disease Prevention and Control, Stockholm, Sweden; 3 Emerging, Food- and Vector-Borne Diseases, European Centre for Disease Prevention and Control, Stockholm, Sweden; 4 Vaccine Preventable Diseases and Immunisation, European Centre for Disease Prevention and Control, Stockholm, Sweden

**Keywords:** Aircraft, coronavirus infection, in-flight transmission, MERS-CoV, Middle East respiratory syndrome coronavirus, travel, RAGIDA

## Abstract

Middle East respiratory syndrome coronavirus (MERS-CoV) causes a potentially fatal respiratory disease. Although it is most common in the Arabian Peninsula, it has been exported to 17 countries outside the Middle East, mostly through air travel. The Risk Assessment Guidelines for Infectious Diseases transmitted on Aircraft (RAGIDA) advise authorities on measures to take when an infected individual travelled by air. The aim of this systematic review was to gather all available information on documented MERS-CoV cases that had travelled by air, to update RAGIDA. The databases used were PubMed, Embase, Scopus and Global Index Medicus; Google was searched for grey literature and hand searching was performed on the EU Early Warning and Response System and the WHO Disease Outbreak News. Forty-seven records were identified, describing 21 cases of MERS that had travelled on 31 flights. Contact tracing was performed for 17 cases. Most countries traced passengers sitting in the same row and the two rows in front and behind the case. Only one country decided to trace all passengers and crew. No cases of in-flight transmission were observed; thus, considering the resources it requires, a conservative approach may be appropriate when contact tracing passengers and crew where a case of MERS has travelled by air.

## Introduction

A novel coronavirus was discovered in 2012 after a patient in Jeddah, Saudi Arabia, died from a severe respiratory disease [1]. The virus, now known as Middle East respiratory syndrome coronavirus (MERS-CoV), has been detected in 27 countries, with 2578 cases reported worldwide as of December 2020, along with 935 deaths (36% crude case fatality rate) [2]. Air travel represents a significant risk for the spread of the disease to other countries.

The *Coronaviridae* are widely distributed among mammals and birds [3], and they are the second most frequent cause of the common cold [4]. This family of viruses was previously thought to cause only mild respiratory diseases in humans, until in 2003 a multi-state outbreak of a respiratory disease led to the identification of the severe acute respiratory syndrome (SARS) coronavirus [5], a betacoronavirus closely related to MERS-CoV. Since December 2019, a new coronavirus, SARS-CoV-2, has led, by 29 December 2020, to a pandemic of over 76 million reported cases and over 1.6 million deaths, triggering unprecedented control measures worldwide with enormous societal and economic effects.

The origin and the transmission of MERS remain poorly understood; however, coronaviruses have been isolated from bats worldwide and MERS-CoV has been detected in camels throughout the Middle East and Africa [6, 7]. Identical genomic sequences of MERS-CoV isolated from humans and their camel contacts have been reported [8], with this animal being the only documented zoonotic source of infection to humans [9]. Human-to-human transmission appears to be limited, but when it does occur, it does so through respiratory droplets and close contact (living with or caring for an infected individual without adequate protection). Most secondary cases occur in family members of the diseased person or in the healthcare setting [9–11].

Around 84% of all MERS cases have been reported from Saudi Arabia [12], and all have been linked to countries in or near the Arabian Peninsula. Seventeen countries outside the Arabian Peninsula have reported travel-associated cases [13] (Table 1). One of these importations culminated in an explosive multi-hospital outbreak in South Korea in 2015, with a total of 186 confirmed cases [14, 15]. The basic reproductive number (*R*_0_) during this outbreak was estimated to be 8.1 before measures were undertaken to control the spread of the disease [16]. However, most studies of the transmission dynamics of MERS show reproductive numbers below or around 1 [9].

**Table 1.** Countries with lab-confirmed MERS cases

**Countries in or near the Arabian Peninsula**
Bahrain, Iran, Jordan, Kuwait, Lebanon, Oman, Qatar, Saudi Arabia, United Arab Emirates and Yemen.
**Countries outside the Arabian Peninsula with travel-associated cases**
Algeria, Austria, China, Egypt, France, Germany, Greece, Italy, Malaysia, Netherlands, Philippines, South Korea, Thailand, Tunisia, Turkey, United Kingdom, United States of America.

The clinical presentation of MERS extends from a mild respiratory disease to a fatal lower respiratory tract infection and the incubation period ranges between 2 and 14 days (average 5 days). Asymptomatic infections have been identified through contact tracing investigations [9] and seroepidemiological studies have found between 9 and 35% of infections to be asymptomatic [17–22], but the role these infections have in the spread of the disease is not yet fully elucidated [23]. Cowling *et al*. [24] suggest that infectiousness might begin 0.4 days before symptom onset, although other studies have shown that patients are not infectious during the incubation period, and only become infectious once symptoms appear [25, 26]. The exact duration of the infectious period is uncertain, although viral excretion from the respiratory tract has been documented throughout the first month of illness, with a higher viral load detected in lower respiratory tract samples [9]. Most MERS cases have occurred in adults, with an average age of 50 years and male predominance [10].

The diagnosis of the disease should be performed by using reverse transcription PCR, real time or conventional, targeting either the upstream of the gene E (UpE gene) or the gene N (UpN gene) as an initial screening test. For confirmation, ORF1b or ORF1a are used [9, 27]. The only current available treatment is symptomatic care since the efficacy of antivirals such as ribavirin and interferon remains controversial [28].

### Transmission of infectious diseases on board aircraft

In 2007, the European Centre for Disease Prevention and Control (ECDC) initiated RAGIDA (Risk Assessment Guidance for Infectious Diseases transmitted on Aircraft) to assist national public health authorities in the EU in the evaluation of the risks associated with the transmission of infectious agents on board aircraft and to advise on measures for containment [29]. This project consists of two parts: the first is a set of guidelines published in 2009 [29], based on a systematic review and expert opinion, which provides a basis for countries to assess in-flight transmission events. The second part includes a set of disease-specific guidance documents [30]. Due to the MERS outbreak, it was decided that a guidance for MERS-CoV would also be produced.

In-flight transmission has been documented for several diseases. The systematic review in 2009 identified 18 cases of on-board transmission from tuberculosis infected persons; 81 infections resulted from influenza infected travellers; 26 passengers were infected during flight by persons with SARS; one person got meningococcal infection; and six people had been infected with measles [29, 31, 32]. Secondary cases in these flights occurred in passengers sitting from two rows away from the index case up to 10 rows away. Additionally, there have been multiple reports of possible and documented in-flight transmission events of the new SARS-CoV-2 [33, 34].

One of the in-flight transmission events of influenza [35] occurred during ground delay, while the engines were shut down for three hours due to a failure during a take-off attempt. This incident resulted in 39 of the 54 passengers infected (72%), and highlights the importance of air circulation on aircraft. Modern airplanes supply fresh air to the cabin from outside during flight, and 50% of that air is recirculated inside the cabin, after passing through high efficiency particulate arresting (HEPA) filters. These filters can remove 99.97% of particles larger than 0.3 μm in diameter from the cabin air. Viruses smaller than 0.3 μm that tend to adhere to particles or form clumps will also be eliminated (MERS-CoV is 0.11–0.14 μm).

The most important public health intervention carried out after a case of any infectious disease with person-to-person transmission potential travels on board aircraft is contact tracing. Contact tracing is the process of identifying people who may have encountered an infected individual, to alert them about the possibility of infection, offer testing for diagnosis and provide prophylactic care, when available. The goal of contact tracing is to interrupt the transmission of the disease and reduce the spread of the infection.

## Objectives

This review aims to gather the available evidence needed to guide health interventions, such as contact tracing, in the event of a case of MERS travelling on aircraft, and to provide a thorough description of cases on aircraft, interventions undertaken, and, if any, in-flight transmission events.

We aim to answer the following primary and secondary questions:

1. Is there any evidence that MERS-CoV has been transmitted to passengers and/or crew on aircraft?
2. Have any interventions been taken after a case of MERS-CoV travelled on aircraft? What were they and what were the outcomes?

## Methods

This systematic review was conducted following the Preferred Reporting Items for Systematic reviews and Meta-analyses (PRISMA) guidelines [36], and the protocol was registered in advance at the International Prospective Register of Systematic Reviews (PROSPERO), an international database created by the Centre of Reviews and Dissemination of The University of York and funded by the National Health Service (NHS) [37].

For the primary question, the study population was defined as all aircraft passengers and crew exposed to a case of MERS on board aircraft. The outcome sought was whether any in-flight transmission to passengers or crew had occurred.

For the secondary question, the study population was defined as all aircraft passengers and crew exposed to a case of MERS on board aircraft, with intervention defined as contact tracing, laboratory testing or authorities informing the study population about the exposure to a MERS case. The outcome sought was a description of the interventions that took place in an attempt to alter the risk of onwards transmission of the disease.

For this review, a ‘case’ refers to a passenger on board aircraft who was diagnosed with MERS-CoV infection (using molecular methods as stated in the WHO case definitions [38]) and who had signs or symptoms compatible with the disease during the flight.

A possible event of in-flight transmission was defined as the detection of any person on board aircraft where a case of MERS had been present, and who was diagnosed with MERS after the flight, who had no other known previous exposure to the virus or risk factors (i.e. direct or indirect contact with camels), and with symptom onset within 14 days of the flight.

### Eligibility criteria

All reports describing a case of MERS who had travelled on aircraft while symptomatic were included in this review. Reports where no public health measures are described after a case of MERS had travelled by aircraft were also included. A report was excluded if the case was asymptomatic during the flight, or if it described an importation or exportation of MERS-CoV between countries, but without specifying that the case had travelled by aircraft. Other exclusion criteria included the setting not being an aircraft, or the disease not MERS.

### Information sources

The electronic literature databases used for retrieval of peer-reviewed articles were Medline (PubMed), Embase, Scopus and Global Index Medicus. Google was used to search for grey literature. Additionally, hand searching was performed on relevant events reported in the EU Early Warning and Response System (EWRS), a communication tool between health authorities in the EU; and on the WHO Disease Outbreak News (DON) section for MERS-CoV. Public health officers were contacted by email to complete information missing from sources.

### Search strategy

Keywords from natural and controlled vocabulary (MeSH terms and Emtree terms) were identified for each component of the study questions, to be used in the electronic literature search. The specific search strategy was created with input from all authors and with support of a Medical Library Specialist, and subsequently peer-reviewed by a second Medical Library Specialist.

No limits were set regarding time coverage, language, type of study design or publication status.

Forward and backward reference checking of the articles selected and all systematic reviews, literature reviews and modelling studies was performed to ensure the identification of all relevant studies.

The initial search was carried out on 18 February 2019, and an alert was set up for all database searches, so notifications of new search results were received up until the final analyses were complete, on 31 May 2019, and eligible studies were retrieved for inclusion.

The search was broadened by including terms related to SARS-CoV on board aircraft to ensure misclassified articles about MERS-CoV were captured, or to gather indirect information on transmission if the number of MERS-CoV-related articles was too low to perform an analysis.

An example of the search strategy, used for PubMed, is included in the box.

**BOX.** Example of search strategy, used for PubMed.

#1	‘Coronavirus’ (Mesh) OR ‘Coronavirus Infections’ (Mesh) OR ‘Middle East Respiratory Syndrome Coronavirus’ (Mesh) OR coronavirus* (TW) OR cov (TW) OR hcov (TW) OR ncov (TW) OR middle east respiratory syndrome (TW) OR ‘hcov-emc’ (TW) OR mers virus* (TW) OR ‘SARS Virus’ (Mesh) OR ‘Severe Acute Respiratory Syndrome’ (Mesh) OR sars (TW) OR ‘Severe Acute Respiratory Syndrome’ (TW) OR ‘Severe Acute Respiratory infection’ (TW) OR ‘sudden acute respiratory syndrome’ (TW)
#2	‘Aerospace Medicine’ (Mesh) OR ‘Aircraft’ (Mesh) OR ‘Aviation’ (Mesh) OR ‘Airports’ (Mesh) OR aircraft* (TW) OR aeroplane* (TW) OR airplane* (TW) OR helicopter* (TW) OR airline* (TW) OR flight* (TW) OR aircrew (TW) OR airflight* (TW) OR aviation (TW) OR airport* (TW) OR aeroport* (TW) OR heliport* (TW) OR ‘aero transport’ (TW) OR ‘air port’ (TW) OR steward (TW) OR stewardess (TW) OR inflight (TW) OR ‘in-flight’ (TW) OR cabin (TW) OR cabins (TW) OR ((‘Travel’ (Mesh) OR travel* (TW) OR transport* (TW) OR transport hub* (TW) OR journey* (TW) OR trip (TW) OR trips (TW)) AND air (TW)) OR ((plane (TW) OR planes (TW)) AND (air (TW) OR travel* (TW) OR ‘Travel’ (Mesh) OR transport* (TW) OR journey* (TW) OR trip (TW) OR trips (TW))) OR ((passenger* (TW) OR crew (TW) OR traveller* (TW) OR traveler* (TW) OR personnel (TW) OR staff (TW) OR pilot* (TW)) AND (flying (TW) OR fly (TW) OR air (TW)))
#3	(#1 AND #2)
#4	(‘time of flight’ (TW) AND spectrometry (TW))
#5	(#3 NOT #4)
Limits: no limits Results: 192 Date of search: 18 February 2019	

The first two search strings (#1 and #2) were combined so that the databases would be searched for papers that included terms from both sections. Any papers with the combination ‘time of flight’ AND ‘spectrometry’ were excluded from the search to avoid identifying studies related to the analytical technique MALDI-TOF, which was not relevant for this study.

### Data management

All results from the database search were uploaded to Endnote v7.8; this tool was used for de-duplication of references, for the initial title and abstract screening process, and for full text retrieval of relevant articles.

### Selection process

For each of the references resulting from the search strategy, an initial title and abstract screening was performed to identify articles relevant to answer the review questions. Titles and abstracts were assessed independently by two reviewers to increase the reliability of the inclusion and exclusion process. For records lacking abstracts, the full text of articles with relevant titles were considered.

At this step, all systematic reviews, literature reviews and modelling studies were included to perform a reference cross-check to validate our search and prevent omissions, even if they were later excluded upon evaluation of the full texts.

For all sources passing the initial abstract screening process, a copy of the full text was retrieved and evaluated by the two reviewers.

Articles were included when both reviewers determined that the article met the inclusion criteria. All disagreements were resolved by consensus.

### Data collection process

Data about the flight, the confirmed MERS case on board, the contact tracing investigation of the flights and any additional public health interventions implemented were extracted using pre-designed tables to systematise the data collection and evaluation. Explicit descriptions of each item were formulated before the extraction. All items retrieved are listed in Table 2. The data extraction process was performed by one author in duplicate to avoid omissions.

**Table 2.** Data extracted from included records

**Flight characteristics**
Flight origin and destination, type of aircraft, date of flight, flight duration, ground delay, number of passengers and crew members on board, HEPA filter function.
**Confirmed case on board**
Country of residence, nationality, age, sex, date of symptom onset, symptoms/signs during flight, date of diagnosis, sample taken and method of diagnosis, seating characteristics on aircraft, travel companion, disease outcome.
**Contact tracing investigation of flight**
Country initiating contact tracing, other countries involved, starting date, duration, definition of contacts, methods used to identify and reach contacts, number of contacts, number of successfully traced contacts, contacts followed for 14 days or more, contacts with respiratory symptoms, contacts tested, contacts with positive test results.
**Other**
Alternative or additional measures taken by authorities, starting date of intervention, duration of intervention, other comments.

When information between different sources regarding the same case conflicted, the information on the source with the highest quality of evidence score (using the Bias Assessment Tool, see below) was considered.

WHO DON reports that only provided an update of a previous mentioned case, but did not report a new case, were regarded as duplicates of the first, but data were updated.

When not reported, flight times were estimated by using Google Maps. When no city of departure/destination was available, capital cities were used. For all MERS cases that travelled by flight while symptomatic, country of flight origin was considered the country of probable exposure.

### Risk of bias in individual studies

Risk of bias assessments were performed on all included records using a modified version of the Bias Assessment Tool (Table 3) developed by Leitmeyer and Adlhoch, 2016 [31]. No studies were excluded based on the score obtained.

**Table 3.** Bias assessment tool

Criteria	Points
Index case classification	
Laboratory confirmation	1
Unspecific clinical presentation or data not provided	0
Secondary case ascertainment	
Laboratory testing of possible cases on flight	2
Syndromic (i.e. MERS-CoV-like illness) or no comprehensive confirmation of all possible cases	1
Not provided	0
Public health interventions	
Contact tracing of flight passengers	2
Other	1
No intervention conducted or not mentioned	0
Timeliness of contact tracing of flight	
Within 1 week	2
Within 2 weeks	1
3 weeks or more	0
Proportion of aircraft contacts followed up	
More than 80% followed up	2
Between 50% and 80% were followed up	1
Less than 50% were followed up or retrospective identification	0
Limitations	
Alternative exposure before flight possible/alternative exposure not addressed	−1
*Resulting evidence levels: 0–4 low, 5–7 medium, 8–9 high.*	

### Data synthesis

All data were collected, summarised and analysed using Microsoft Excel (2016).

## Results

### Identified records

**Fig. 1.**
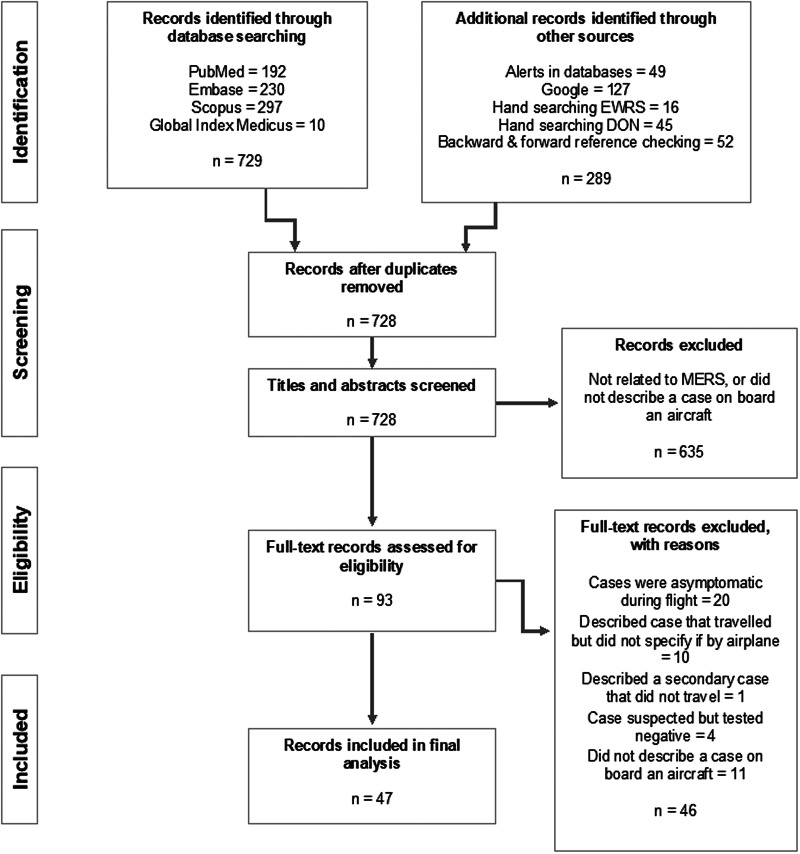
Flow diagram of study selection process.

A total of 47 records (18 peer-reviewed articles, 9 EWRS notifications and 20 WHO DON reports) describing 21 cases of MERS who had travelled on aircraft while symptomatic were identified for inclusion in this review. The search of online medical databases provided a total of 729 references, plus 49 additional records obtained by the set-up alerts. Another 240 records were identified through hand searching, Google searching and reference checking, for a total of 1018 records. After de-duplication, 728 records remained for title and abstract screening. Of these, 635 records were discarded because they did not meet the inclusion criteria. The full text of the 93 remaining records was evaluated in more detail, and 47 were deemed relevant for this review. Reasons for exclusion are described in Figure 1.

None of the records included described all items sought. All records reported country of flight origin and destination, although 28 (60%) did not report city of departure and 17 (36%) did not report city of destination. None of the records mentioned any ground delay or the HEPA filter function.

One article mentioned the type of aircraft, one mentioned the exact total flight duration, two mentioned the total number of passengers and crew members on board and one mentioned the sitting characteristics of the case on board. Twenty-nine records mentioned a contact tracing investigation was done on the case's flight. Communication with three public health officers did not reveal new information regarding the included cases.

Of the 47 records, 26 were classified as having a low level of evidence, most of which were WHO DON reports and EWRS notifications, due to the limited amount of information contained in them; 17 records obtained a medium score, and four peer-reviewed articles got a high score (Appendix 1).

### Cases of MERS that travelled by aircraft

The 21 cases described boarded 31 flights while symptomatic. Three of the cases were transported to another country by air ambulances. No secondary cases of in-flight transmission were identified.

**Fig. 2.**
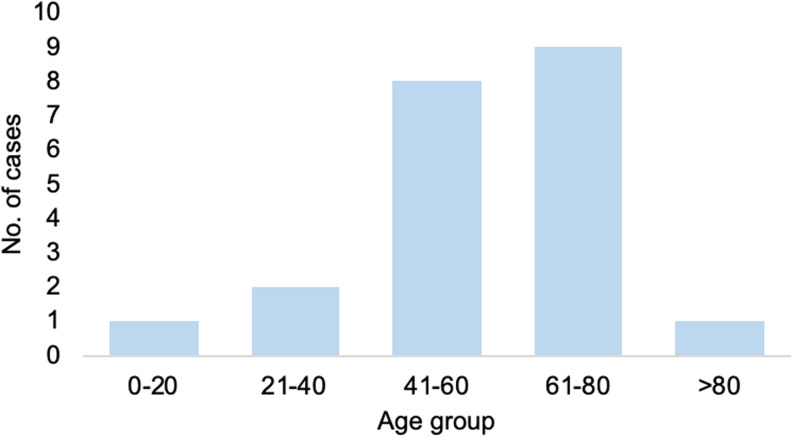
Number of MERS cases per age group.

**Fig. 3.**
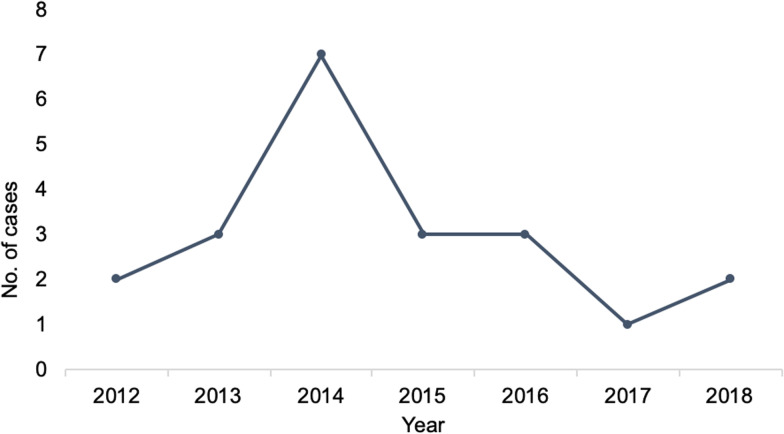
Number of MERS cases that travelled by flight per year.

Eighteen cases were male (86%). The median age was 57.5 years (range 18–85 years) (Fig. 2). Thirteen cases boarded only one flight during their trip, seven boarded two and one took four. Most cases travelled in 2014 (Fig. 3), and the average total flight time (sum of all flights boarded by an individual) was 7.4 h. The shortest single flight duration was 1.3 h and the longest was 9.5 h. Most cases had probably been exposed in Saudi Arabia (12 cases). Other countries of flight origin were Jordan (1), Kuwait (1), Oman (2), Qatar (2), South Korea (1) and United Arab Emirates (1).

Eight cases were residents of Saudi Arabia. Other countries of residence included Qatar, United Arab Emirates, United Kingdom, Italy, Netherlands, South Korea, Oman, Kuwait and Malaysia. Six cases travelled with family members.

**Fig. 4.**
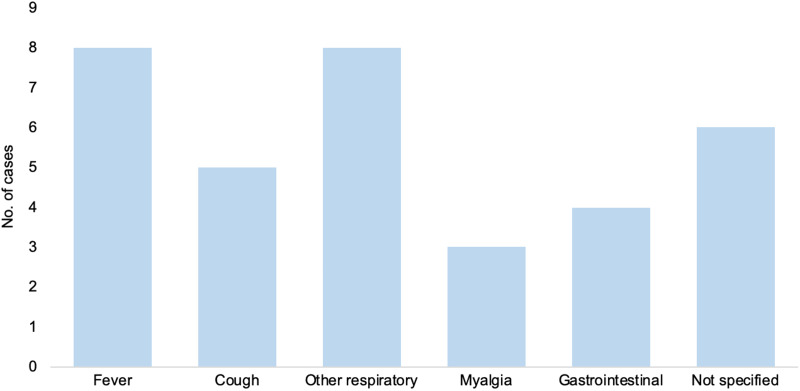
Number of cases that presented each symptom during the flight. Note that one case may present more than one symptom.

**Fig. 5.**
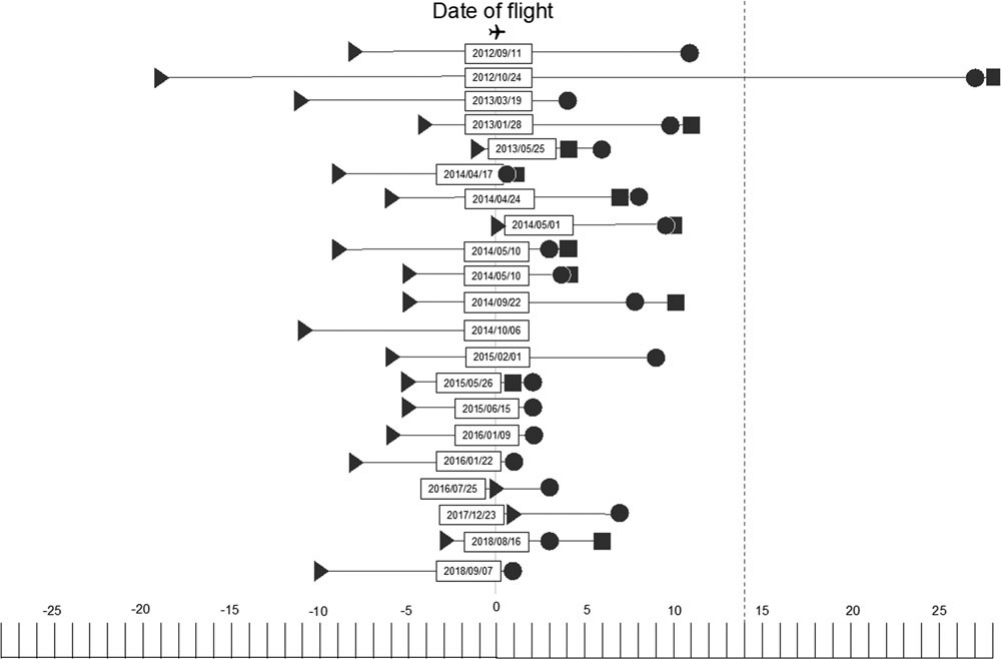
Days between onset of symptoms (triangles), flight date (yyyy/mm/dd), diagnosis (circles) and start of contact tracing (squares). Scale refers to number of days before or after the flight. Dotted line marks 14 days after flight.

The most common symptoms present during flight were fever and respiratory symptoms other than cough (Fig. 4). The three cases transported by air ambulance had a severe disease and were intubated. On average, the date of the flight was 6.2 days (range 0–19) after the date of symptom onset, corresponding to the point in the course of infectiousness the flight occurred (Fig. 5).

The outcome of the disease was not mentioned for all cases (70.2% of the 47 records had no information), however, at least three died and nine were discharged from the hospital as cured. The observed case fatality rate among the 12 cases with known outcome was 33%, and among all 21 cases was 14%.

### Contact tracing investigations

Out of the 47 records included in the review, 29 stated that a contact tracing investigation had been carried out on the case's flight, and of those, 19 described the contact tracing results. Contact tracing of the passengers and crew on board the aircraft was carried out for at least 17 cases and 24 flights; it was not mentioned whether contact tracing had been carried out for the remaining four cases. At least 18 countries are mentioned to have been involved in a contact tracing investigation: Austria, China, Germany, Greece, Italy, Malaysia, the Netherlands, Oman, Philippines, Poland, Qatar, Saudi Arabia, South Korea, Taiwan, Thailand, Turkey, the United Kingdom and the United States of America.

Cases were diagnosed an average of six days after they had travelled on aircraft. Contact tracing investigations were initiated an average of 0.5 days after the case was diagnosed with MERS, that is, either on the same day of diagnosis or just before, when MERS-CoV infection was suspected (Fig. 5). The average reported duration of contact tracing investigations was 13.5 days.

‘Contacts’ for contact tracing investigations were most commonly defined as the passengers sitting in the same row as the case and in the two rows in front and behind (known as the two-row rule). Contact definitions for passengers and crew per country performing the investigation are described in more detail in Table 4. A total of 374 contacts for nine of the cases were tested. The most common criterion for testing was the development of symptoms during the 14-day period following the flight, however, for cases 8 and 15, all contacts that could be tested underwent testing (Table 6). The most common method was nose and throat swabbing for PCR analysis, but 218 contacts of case 8 underwent serological testing. No contacts were reported positive for MERS-CoV.

**Table 4.** Contact definition by country and year

Country	Year	Definition for passengers	Definition for crew
Germany (air ambulance)	2012	Contact tracing of healthcare workers including those during transport to the hospital.	.
UK	2013	Two-row rule.	.
Italy	2013	Two-row rule.	All crew
Greece	2014	Two-row rule.	.
UK	2014	Two-seat radius around case prioritised, rest of passengers only tested and followed if they became symptomatic.	.
USA	2014	All passengers and crew (for all emerging diseases).	All crew
Netherlands	2014	Within three rows of case.	.
China	2014	Close contacts: two-row rule; other contacts: all other passengers.	.
Philippines	2015	Category A: passengers in surrounding three rows; category B: in surrounding three rows only transiting in Philippines; category E: all other passengers. (categories C and D were regarding contacts outside the flight).	.
China	2015	Close contacts: passengers in the adjacent two rows; other contacts: all other passengers on flight.	All crew
Thailand	2015	Passengers in the two rows surrounding case. Low risk: contact further than 1 m.	High risk: interaction closer than 1 m; low risk: contact further than 1 m
UK	2018	Three rows in front and behind.	.
South Korea	2018	Passengers sitting near case.	All crew

The most widely used method for identifying contacts was requesting the passenger manifest and contact details from the airline. Contacts were most commonly reached by phone (Table 5). Some countries set up a hotline so passengers of the flight could call if they developed symptoms.

**Table 5.** Methods used to identify and reach contacts by country

Country	Methods used to identify contacts	Methods used to reach contacts
Germany (air ambulance)	Requested healthcare workers to report contact with the patient during air transport to hospital.	Questionnaire given to fill in.
Italy	Requested contact details from airline.	.
Greece	Requested contact details from airline.	Phone.
UK	Requested contact details from airline; requested through press release that passengers of the flight call a health phone service (hotline).	Phone.
Austria	Requested contact details from airline	Crew data sent to WHO to communicate to Qatar.
China	Passenger list provided by WHO; airline provided seating plan and contact details; travel agency provided tour member list; hotline set up and case's travel details published.	Some contacts had an initial interview in person and were monitored by phone; others called hotline.
Philippines	.	Interviewed in person.
USA	Ordered passenger manifest from airline, use of federal databases (i.e. border control), custom declaration forms, contact with Public Health England for details of Riyadh-London passengers.	Phone, email, letter, interviewed in person. Crew contacted by airline.
Thailand	Requested passenger details from airline.	Phone, located at address, voluntarily reported and interviewed in person.

**Fig. 6.**
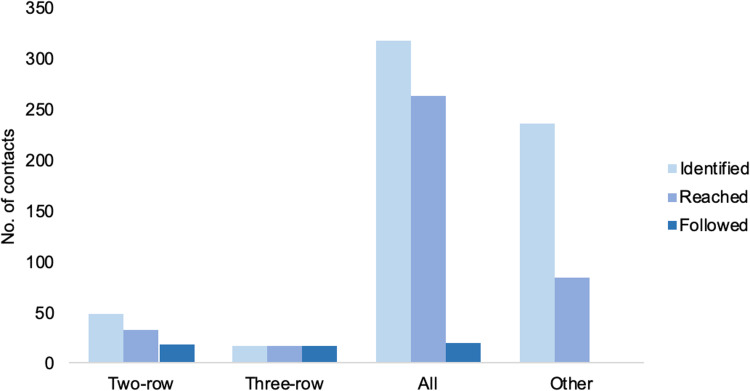
Average number of contacts identified, reached and followed by contact definition implemented.

The median and the mean number of contacts identified per flight were 24 and 109.9, respectively. This varied widely between cases (range 9–561), depending on the contact definition used (two or three rows, whole plane) and on the number of flights boarded by each case. In most contact tracing investigations, more than 50% of the identified contacts were reached and followed for the 14-day maximum incubation period of MERS-CoV. This again was dependent primarily on the definition of contacts used; the most successful interventions (records that mention that 100% of the contacts were reached and followed for 14 days) were observed when a two- or three-row approach was taken, and was the least successful when more complicated definitions were used, such as dividing contacts into several categories (Fig. 6). Contact tracing investigations are described in detail in Table 6.

**Table 6.** Summary of contact tracing investigations of flights

Sources	Case	Flights boarded	Identified contacts	Contacts reached (%)	Contacts followed 14 days (%)	Contacts that developed symptoms	Contacts tested	Positive contacts
EWRS 23 September 2012 [41], Bermingham [42], Pebody [43], DON 23 September 2012 [44]	**1**	1	.	.	.	.	.	.
EWRS 23 November 2012 [41], Buchholz [45]	**2**	1	.	.	.	.	.	.
EWRS 23 November 2012 [41], Reuss [46], DON 26 March 2013 [47]	**3**	1	.	.	.	.	.	.
EWRS 8 February 2013 [41], HPA[48], DON 11 February 2013 [49]	**4**	1	20	11 (55)	11 (55)	2	1	0
EWRS 31 May 2013 [41], Puzelli[50], DON 1 June 2013 [51]	**5**	2	9	9 (100)	9 (100)	0	.	.
EWRS 18 April 2014 [41], Tsiodras[52], DON 20 April 2014 [53]	**6**	2	12	.	.	.	.	.
EWRS 2 May 2014 [41], Bialek [54], Parry-Ford [39], Regan [55], Lippold [56], DON 5 May 2014[57]	**7**	2	269	144 (53)*	42 (15)	16	3	0
EWRS 2 May 2014 [41], Bialek [54], Parry-Ford [39], Regan [55], Lippold [56], DON 14 May 2014[58]	**8**	4	561	450 (80)*	3 (0.5)	35	230	0
EWRS 14 May 2014 [41], Kraaij [59], Mollers [60], DON 15 May 2014 [61]	**9**	2	17	17 (100)	17 (100)	2	17	0
EWRS 14 May 2014 [41], Kraaij [59], Mollers [60], DON 16 May 2014 [62]	**10**	2	17	17 (100)	17 (100)	2	17	0
EWRS 30 September 2014 [41], Kwok-ming [63], DON 2 October 2014 [64]	**11**	2	43	43 (100)	43 (100)	1	1	0
DON 24 October 2014 [65]	**12**	1	.	.	.	.	.	.
Racelis[66], DON 13 February 2015 [67]	**13**	1	237	85 (35)	.	0	85	0
Wu [68], Kang[69], DON 30 May 2015 [70]	**14**	1	27	27 (100)	6 (22)	0	6	0
Plipat [71], DON 20 June 2015 [72]	**15**	1	89	89 (100)	26 (29)	0	14	0
DON 26 January 2016 [73]	**16**	1	.	.	.	.	.	.
DON 29 January 2016 [74]	**17**	1	.	.	.	.	.	.
DON 26 August 2016 [75]	**18**	1	.	.	.	.	.	.
DON 8 January 2018 [76]	**19**	1	.	.	.	.	.	.
EWRS 23 August 2018 [41], DON 31 August 2018 [77]	**20**	1	18	17 (94)	.	.	.	.
DON 12 September 2018 [78]	**21**	2	.	.	.	.	.	.
		**Total**	1319	909	174	58	374	0
		**Mean**	109.92	82.64	19.33	5.8	41.5	0
*Includes people who rejected interview		**Median**	24	27	17	2	14	0

Cases 1−3 in this review (Table 6) were transported via air ambulance, and information about the people present during the transport, as well as whether contact tracing was carried out for those flights, was not reported.

The most important factor that delayed the possibility of conducting an adequate contact tracing of the flight mentioned by authors, was the unavailability of all passengers' contact details, since many airlines do not demand these details in all situations for booking tickets.

### Additional or alternative health interventions

The most common additional health intervention described in the reports included contact tracing of healthcare workers, family members and other contacts of the case not present on the flight, such as co-workers or bus passengers, which was mentioned in 21 records.

Other additional or alternative interventions carried out by national public health authorities included: press releases to alert passengers and crew about a possible exposure to MERS-CoV and to inform on what measures to take, such as going to a health care professional if they develop any symptoms (mentioned in eight reports); setting up a hotline for passengers on the flight to be able to reach authorities (one report); and communication with international public health authorities of countries whose nationals had been on board the flight (seven reports). One of the countries also decided to evaluate the psychological stress generated by the contact tracing investigation on the passengers of the flight, and concluded that contact tracing seems to be a stressful event for passengers.

### Other findings

During the screening process, 16 additional records were found describing eight cases of MERS that had travelled by aircraft before symptom onset. For two of these cases, contact tracing of the flights was carried out and no secondary cases related to these flights have been reported by any of the involved countries (Appendix 2).

## Discussion

In this review, no evidence of secondary transmission of MERS-CoV on board aircraft was found among the 21 cases included in the study. To the authors' knowledge, there have been no secondary cases reported from flights worldwide, in contrast with at least 26 secondary in-flight transmission events documented during the multi-state outbreak of SARS, and multiple reports of SARS-CoV-2 transmission. MERS-CoV seems to not have acquired the ability of sustained transmission between humans outside of healthcare settings or households – situations where close contact or aerosol generating procedures are frequent.

The demographic characteristics of the reported cases who had travelled on board aircraft (male predominance and median age of 57 years) were not markedly different from those of all globally reported cases. Most cases were probably exposed in Saudi Arabia, the country with the highest number of MERS-CoV cases worldwide. The finding that most cases travelled during 2014 also coincides with an outbreak in Saudi Arabia during that period [2]. The low case fatality rate observed in this study could be explained by missing data on the disease outcome from most reports, or bias due to severely ill individuals being less likely to travel.

An important factor to consider when deciding who to trace, is the movement of passengers around the aircraft. Any passenger that changed seats may therefore have to be included (or excluded) in the contact tracing investigation. The case may also have interacted with passengers sitting on further rows during the flight, and proximity during boarding or disembarking, while hard to ascertain, may be relevant.

It was encouraging to find that most contact tracing investigations were initiated soon after the cases were diagnosed with MERS-CoV. This highlights that countries involved in the reported events have appropriate guidelines in place and have the capacity to respond timely to these events. From this review we cannot conclude if this is the case for other countries.

The aim of this review was to gather all available information on MERS-CoV cases that have travelled by aircraft and any evidence of in-flight transmission events, with the goal of producing an update of the RAGIDA project including a chapter on MERS-CoV. Having no transmission events during any flight hinders the possibility of making an assertive recommendation on whether to trace a limited number of rows or the whole plane when a case of MERS-CoV travels by aircraft. Since no transmission has been observed either on rows around the case or on further rows, it is not possible to know how far away from the case the infection is likely to spread. The lack of in-flight transmission observed in this study suggests that a more conservative approach, with fewer rows traced, may be adequate, also considering the large resources needed for conducting contact tracing investigations [29, 39].

### Limitations

The most significant limitation was the scarce amount of data regarding the flights and the contact tracing investigations from many of the records included. However, records were not excluded based on the quality or quantity of the information given in order to extract the largest possible amount of information and to avoid any omissions. Although experts were contacted regarding missing data, no further information that could have improved the analysis was obtained.

Furthermore, for 15 of the 21 cases included more than one source of information was identified. Two cases were mentioned each in six different records. Incorporating all the information from several sources into one set of results, while avoiding making incorrect assumptions, was challenging but has, in our opinion, resulted in the best available evidence.

Although it was not expected that every record identified would report all items sought in the review, none of the sources mentioned whether there was any ground delay of the flights, or whether it was known if the HEPA filter was functioning adequately. Since these factors have been important in previous in-flight transmission, future case reporting should consider them to support learning and decision making regarding contact tracing. Similarly, only two records mentioned the total number of passengers and crew members on board; one mentioned the exact duration of the flight, and one mentioned the seating characteristics of the case. We consider the items used for the review to be useful for future contact tracing data collection and reporting (shown in Table 2).

### Contact tracing guidance

On 26 November 2019, ECDC organised a meeting to discuss the results of this systematic review and determine the most adequate course of action to take whenever a case of MERS-CoV has travelled by aircraft. The guidance has been published online [40]. In the guidance, contacts have been defined as passengers seated two seats in all directions around the index case, crew members serving the section of the aircraft where the index case was seated and persons who had close contact with the index case e.g. travel companions or persons providing care. The recommended algorithm to follow when contact tracing can be initiated within 14 days after the flight, is to perform a full contact tracing and follow-up of contacts for a duration of 14 days after the flight took place. If contact tracing is initiated between 14 and 28 days after the flight, contacts may be contacted once to ask if symptoms have developed. When more than 28 days have passed since the flight, no contact tracing has been recommended.

## Data Availability

Data that support the findings of this study are available as described under the public access to ECDC documents page https://www.ecdc.europa.eu/en/about-us/document-request

## APPENDIX 1

**Table A1.** Summary of level of evidence for MERS-CoV in-flight transmission per record

Study	Index case classification	Secondary case ascertainment	Public health intervention	Timeliness of contact tracing of flight	Aircraft contacts followed-up	Alternative exposures	Total	Evidence level
EWRS 23 September 2012	1	2	1	0	0	0	4	Low
EWRS 23 November 2012	1	0	1	0	0	0	2	Low
EWRS 8 February 2013	1	0	0	0	0	0	1	Low
EWRS 31 May 2013	1	2	2	2	0	0	7	Medium
EWRS 18 April 2014	1	0	2	0	0	0	3	Low
EWRS 2 May 2014	1	0	2	0	0	0	3	Low
EWRS 14 May 2014	0	0	2	0	0	0	2	Low
EWRS 30 September 2014	1	0	0	0	0	0	1	Low
EWRS 23 August 2018	1	0	2	0	0	0	3	Low
Bermingham, 2012	1	0	0	0	0	0	1	Low
Pebody, 2012	1	0	1	0	0	0	2	Low
Buchholz, 2013	1	0	1	0	0	0	2	Low
Puzelli, 2013	1	0	2	2	2	0	7	Medium
HPA, 2013	1	2	2	2	2	−1	8	High
Bialek, 2014	0	2	2	2	0	0	6	Medium
Kraaij, 2014	1	2	2	2	2	0	9	High
Reuss, 2014	1	0	1	0	0	0	2	Low
Tsiodras, 2014	1	0	2	2	0	0	5	Medium
Kwok-ming, 2015	1	2	2	1	0	0	6	Medium
Mollers, 2015	1	2	2	0	2	0	7	Medium
Parry-Ford, 2015	1	2	2	1	2	0	8	High
Racelis, 2015	1	2	2	0	2	0	7	Medium
Wu, 2015	1	2	2	2	2	0	9	High
Kang, 2016	0	2	2	2	1	0	7	Medium
Regan, 2016	0	0	2	2	2	0	6	Medium
Lippold, 2017	0	2	2	0	2	0	6	Medium
Plipat, 2017	1	2	2	0	2	0	7	Medium
DON 23 September 2012	1	0	0	0	0	0	1	Low
DON 11 February 2013	1	0	1	0	0	0	2	Low
DON 26 March 2013	1	0	0	0	0	0	1	Low
DON 1 June 2013	1	0	0	0	0	0	1	Low
DON 20 April 2014	1	0	2	2	0	0	5	Medium
DON 5 May 2014	1	0	2	0	0	0	3	Low
DON 14 May 2014	1	0	2	2	0	0	5	Medium
DON 15 May 2014	1	0	0	0	0	0	1	Low
DON 16 May 2014	1	0	2	0	0	0	3	Low
DON 2 October 2014	1	0	2	2	0	0	5	Medium
DON 24 October 2014	1	0	2	0	0	0	3	Low
DON 13 February 2015	1	0	2	2	0	0	5	Medium
DON 30 May 2015	1	0	1	0	0	0	2	Low
DON 20 June 2015	1	0	1	0	0	0	2	Low
DON 26 January 2016	1	0	0	0	0	0	1	Low
DON 29 January 2016	1	0	2	2	0	0	5	Medium
DON 26 August 2016	1	0	1	0	0	0	2	Low
DON 08 January 2018	1	0	2	1	0	0	4	Low
DON 31 August 2018	1	0	2	1	0	0	4	Low
DON 12 September 2018	1	0	2	2	0	0	5	Medium
Average	1	1	1	1	0	0	4	Low

Index case classification: whether or not it was confirmed using laboratory testing; secondary case ascertainment: laboratory testing of possible cases on flight; public health interventions: if contact tracing was performed; timeliness of contact tracing: whether it was carried out within one, two or three weeks or more; proportion of aircrafts contacts followed up: 80%, 50% or less; limitations: whether alternative exposures before flight were considered.

## APPENDIX 2

**Table A2.** Additional MERS cases found, that travelled while asymptomatic

Case	Flight origin	Flight destination	Date of flight	Country of residence	Age	Sex	Date of symptom onset	Date of diagnosis	Contact tracing
1	Dubai, UAE	France	17 April 2013	France	64	M	22 April 2013	7 May 2013	No
2	Qatar	Tunisia	28 May 2013	Tunisia	66	M	1 May 2013	5 August 2013	No
3	Jeddah, Saudi Arabia	Kuala Lumpur, Malaysua (via Istanbul)	28 March 2014	Malaysia	54	M	4 April 2014	14 April 2014	Yes
4	Abu Dhabi, UAE	Germany	10 February 2015	Germany	65	M	12 February 2015	.	No
5	Doha, Qatar	Seoul, South Korea	4 May 2015	South Korea	68	M	11 May 2015	20 May 2015	.
6	Singapore	Manila, Philippines	25 June 2015	Finland	36	M	30 June 2015	4 July 2015	Yes
7	Abha, Saudi Arabia	Vienna, Austria (via Cairo)	4 September 2016	Saudi Arabia	67	M	6 September 2016	8 September 2016	No
8	Saudi Arabia	Lebanon	11 June 2017	Saudi Arabia	39	M	8 June 2017	16 June 2017	No
